# Association of the serological status of rheumatoid arthritis patients with two circulating protein biomarkers: A useful tool for precision medicine strategies

**DOI:** 10.3389/fmed.2022.963540

**Published:** 2022-10-28

**Authors:** Cristina Ruiz-Romero, Patricia Fernández-Puente, Lucía González, Anna Illiano, Lucía Lourido, Rocío Paz, Patricia Quaranta, Eva Perez-Pampín, Antonio González, Francisco J. Blanco, Valentina Calamia

**Affiliations:** ^1^Unidad de Proteómica, Grupo de Investigación de Reumatología (GIR), Instituto de Investigación Biomédica de A Coruña (INIBIC), Complexo Hospitalario Universitario de A Coruña (CHUAC), Sergas, Universidade da Coruña (UDC), A Coruña, Spain; ^2^Centro de Investigación Biomédica en Red de Bioingeniería, Biomateriales y Nanomedicina (CIBER-BBN), Madrid, Spain; ^3^Centro de Investigaciones Científicas Avanzadas (CICA), Universidad de A Coruña (UDC), A Coruña, Spain; ^4^CEINGE—Advanced Biotechnology, Naples, Italy; ^5^Department of Chemical Sciences, University of Naples Federico II, Naples, Italy; ^6^Laboratorio de Investigación 10 and Rheumatology Unit, Instituto de Investigación Sanitaria (IDIS), Hospital Clínico Universitario de Santiago (CHUS), Santiago de Compostela, Spain; ^7^Grupo de Investigación de Reumatología y Salud (GIR-S), Departamento de Fisioterapia, Medicina y Ciencias Biomédicas, Facultad de Fisioterapia, Universidade da Coruña (UDC), A Coruña, Spain

**Keywords:** rheumatoid arthritis, biomarker, haptoglobin, orosomucoid 1, multiple reaction monitoring

## Abstract

Rheumatoid arthritis (RA) is an autoimmune disease characterized by chronic inflammation of the joints and presence of systemic autoantibodies, with a great clinical and molecular heterogeneity. Rheumatoid Factor (RF) and anti-citrullinated protein antibodies (ACPA) are routinely used for the diagnosis of RA. However, additional serological markers are needed to improve the clinical management of this disease, allowing for better patient stratification and the desirable application of precision medicine strategies. In the present study, we investigated those systemic molecular changes that are associated with the RF and ACPA status of RA patients. To achieve this objective, we followed a proteomic biomarker pipeline from the discovery phase to validation. First, we performed an iTRAQ-based quantitative proteomic experiment on serum samples from the RA cohort of the Hospital of Santiago de Compostela (CHUS). In this discovery phase, serum samples from the CHUS cohort were pooled according to their RF/ACPA status. Shotgun analysis revealed that, in comparison with the double negative group (RF–/ACPA–), the abundance of 12 proteins was altered in the RF+/ACPA+ pool, 16 in the RF+/ACPA– pool and 10 in the RF-/ACPA+ pool. Vitamin D binding protein and haptoglobin were the unique proteins increased in all the comparisons. For the verification phase, 80 samples from the same cohort were analyzed individually. To this end, we developed a Multiple Reaction Monitoring (MRM) method that was employed in a comprehensive targeted analysis with the aim of verifying the results obtained in the discovery phase. Thirty-one peptides belonging to 12 proteins associated with RF and/or ACPA status were quantified by MRM. In a final validation phase, the serum levels of alpha-1-acid glycoprotein 1 (A1AG1), haptoglobin (HPT) and retinol-binding protein 4 (RET4) were measured by immunoassays in the RA cohort of the Hospital of A Coruña (HUAC). The increase of two of these putative biomarkers in the double seropositive group was validated in 260 patients from this cohort (*p* = 0.009 A1AG1; *p* = 0.003 HPT). The increased level of A1AG1 showed association with RF rather than ACPA (*p* = 0.023), whereas HPT showed association with ACPA rather than RF (*p* = 0.013). Altogether, this study has allowed a further classification of the RA seropositive patients into two novel clusters: RF+A1AG+ and ACPA+HPT+. The determination of A1AG1 and HPT in serum would provide novel information useful for RA patient stratification, which could facilitate the effective implementation of personalized medicine in routine clinical practice.

## Introduction

Rheumatoid arthritis (RA) is an autoimmune pathology mainly characterized by chronic inflammation of the diarthrodial joints and other extra-articular tissues. Effective treatments currently available are few and present serious side effects. In the late stages of the disease, the persistent symmetric and erosive synovitis leads to structural damage and permanent disability (1). Once the osteochondral lesion appears, the damage is irreversible, which turns of paramount importance the early diagnosis of the pathology and the establishment of an effective treatment as soon as possible. However, RA is also characterized by heterogeneous clinical manifestations and a very variable course, which makes it difficult to select effective strategies. Although it currently exists a plethora of drugs that have markedly improved the management of RA and the patient's quality of life, still around 20–40% of patients do not respond to treatment and the mechanisms that lead to this resistance are not yet known (2). Therefore, one of the most relevant medical needs in the field of RA is related to the stratification of patients and the establishment of precision medicine strategies (3, 4).

RA is also characterized by the presence of specific autoantibodies in the patient's sera, which are used in the clinical routine as biomarkers for disease diagnosis. The most representative is Rheumatoid Factor (RF), which was firstly described in RA as it is present in around 80% of the patients (5). RF is an antibody that binds the Fc region of Immunoglobulin G (IgG), forming immune complexes that contribute to the disease process. RF is present in other rheumatic diseases, such as Sjögren's syndrome, and its elevated levels have been associated with more persistently active synovitis, more joint damage, greater eventual disability and arthritis (6). Nevertheless, the specificity of RF for established RA is quite low (between 60 and 70%) (7) and studies on its association with response to different treatments have displayed controversial results (8). The second most characteristic autoantibodies in RA are those originated against citrullinated proteins, or ACPA. Although they are much more specific than RF (96%), their diagnostic sensitivity in early arthritis is 57% (9). Furthermore, up to 30% of RA patients never develop these autoantibodies (10). These considerations point out the need to identify novel molecular biomarkers that could aid in the early diagnosis and stratification of patients with RA.

Interestingly, the major role of biomarkers in RA can be easily objectified by comparing the diagnostic criteria before and after 2010. From 1987, and during the next 23 years, the only ACR criteria biomarker was RF, whereas in the last ACR/EULAR 2010 criteria for the early diagnosis of RA three serological tests were added (ACPA, ESR and CRP) (11) and this demonstrated to improve significantly the clinical management of the disease. However, in the era of personalized medicine, mostly subjective criteria continue to be the basis of the RA diagnosis, evaluation and treatment (12). In addition to difficulties for early diagnosis, this holds predicting disease course and response to treatment very imprecise.

Quantitative proteomics has demonstrated its power for the identification of novel RA protein markers (13). In this field, proteomics studies have also contributed to increase the knowledge of disease etiopathogenesis. One the main goal of proteomics investigations remains the discovery of promising biomarkers for patient stratification, which would undoubtedly have a tremendous impact in improving the clinical management of RA patients (14, 15). Identifying specific phenotypes and endotypes can inform both prognosis and guide therapeutic development for RA disease, with the potential of positively impacting patient care. Although clinical phenotypes are the most common method of subgrouping RA, the importance of identifying endotypes for targeted treatment has gained much attention particularly from the point of view of drug discovery, where identifying the right target is key for success. At the moment, there are few positive examples of RA endotyping other than RF and ACPA. Dennis et al. described four potential endotypes related to drug response: (i) lymphoid (B and plasma cell dominant), (ii) a myeloid (macrophage dominant), (iii) a fibroid, and (iv) a low inflammatory phenotype (16). The knowledge of molecular RA endotypes evidenced with sensitive techniques such as high-throughput proteomics, could be used to target specific subgroups of patients that could benefit from a variety of targeted preventive treatment strategies allowing personalized interventions in RA (17). In the present work, we aimed to carry out a pipeline based on quantitative proteomics tools to discover, verify and validate circulating proteins that are associated with the presence in serum of RF and ACPA and may therefore have potential for the stratification of RA patients and the application of precision medicine strategies based on these molecular signatures.

## Materials and methods

### Serum samples

**Figure 1:**
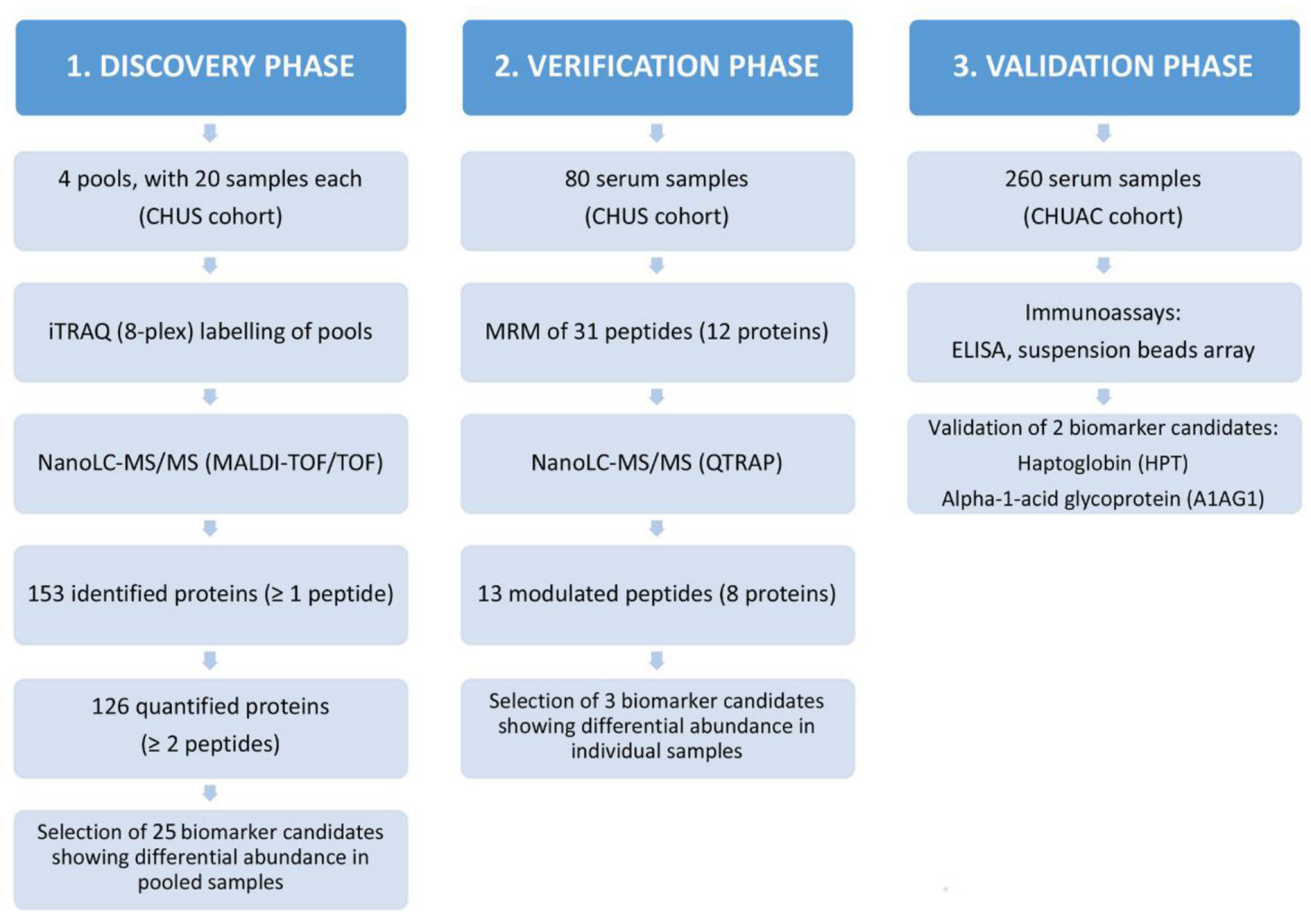
Experimental design of this study. Schematic representation of the proteomic biomarker workflow employed in this work for the identification of RA biomarkers and the results obtained hereof.

The experimental design of this work was structured in three phases, as illustrated in Figure 1: discovery, verification and validation. The study employed serum samples from patients diagnosed with RA according to the criteria of the American College of Rheumatology (18). The 80 serum samples analyzed in the discovery and verification phases belong to a cohort from the Rheumatology Unit of the Complejo Hospitalario Universitario de Santiago de Compostela (CHUS), whereas the 260 samples included in the validation phase correspond to a cohort from the Hospital Universitario de A Coruña (CHUAC). Age, gender, ACPA and RF status were recorded for all patients. These samples were divided into four distinct sub-groups, according to their RF and ACPA values: RF+/ACPA+, RF+/ACPA–, RF–/ACPA+, and RF–/ACPA–. In the CHUS cohort, each group was composed by 20 serum samples, whereas the 260 samples of the CHUAC cohort had the following distribution: 110 FR+/ACPA+ (43.1%); 50 RF+/ACPA– (19.2%); 74 RF–/ACPA– (28.5%) and 26 RF–/ACPA+ (9.2). The demographic characteristics of the samples included in this study are detailed in Table 1.

**Table 1:** Basic demographic and clinical characteristics of the subjects included in this study.

	**Group**	**n**	**RF (IU/ml)**	**Anti-CCP (U/ml)**	**Sex (F/M)**	**Age**
LC-MS/MS (discovery and verification)	RF+/ACPA+	20	ND	ND	15/5	62.6 ± 11.6
	RF+/ACPA–	20	ND	ND	16/4	62.5 ± 15.8
	RF–/ACPA+	20	ND	ND	18/2	59.1 ± 12.4
	RF–/ACPA–	20	ND	ND	15/5	58.7 ± 14.8
Immunoassays (validation)	RF+/ACPA+	110	298.89 ± 504.84	288.77 ± 357.85	78/32	60.05 ± 12.91
	RF+/ACPA–	50	189.11 ± 287.36	11.39 ± 8.15	37/13	53.08 ± 16.29
	RF–/ACPA+	26	20 ± 0	220.43 ± 172.45	22/4	55.65 ± 13.64
	RF–/ACPA–	74	19.99 ± 0.12	8.14 ± 7.69	54/20	56.5 ± 14.7

ND: not detected.

The work was carried out in accordance with the ethical principles of the Declaration of Helsinki and good clinical practice. All patients read and signed the informed consent in which were objectified the use of data/samples for basic investigation. The research protocol was revised and approved by the Local Ethics Committee (Comité de Ética de la Investigación de A Coruña—Ferrol, Galicia, Spain).

### Proteomic biomarker pipeline

In this study, we followed a classical proteomic biomarker pipeline (Figure 1). We started with a shotgun analysis by multiplexed isobaric tagging technology for relative quantitation (iTRAQ) in a reduced number of pooled samples (80 sera grouped in 4 pools). Then, we moved on to the verification analysis of altered proteins by targeted MS-based assays (multiple reaction monitoring analysis, MRM). In this step, we analysed the same 80 samples individually in order to confirm the results obtained in the discovery phase. Finally, we performed the validation analysis by immunoassays on a second cohort of 260 RA patients.

### Shotgun proteomics by iTRAQ labelling and LC-MS/MS analysis

The shotgun proteomic analysis of the discovery phase was performed on 80 sera from the CHUS cohort. The 20 samples per group to be compared (RF+/ACPA+, RF+/ACPA–, RF–/ACPA+ and RF–/ACPA–) were pooled at equal amounts to reduce interindividual variability. Then, these four pools were analyzed in duplicate by 8-plex iTRAQ-based quantitative proteomic approach (Isobaric tags for relative and absolute quantitation, Sciex). The experimental workflow is illustrated in Supplementary Figure S1.

First, the pooled samples were depleted from the top-14 most abundant serum proteins by affinity chromatography, using a MARS Hu-14 column mounted on a 1,200 series HPLC with UV detector set at 280 nm (Agilent Technologies). Then, protein concentration was determined by NanoDrop spectrophotometer (Thermo Scientific) and 50 μg of each pool were digested with trypsin following standard protocols. The resultant peptide mixtures were differentially labeled using the iTRAQ reagents (Supplementary Figure S1), according to the manufacturer's instructions (Sciex) and a protocol previously described by our group (19). Briefly, aliquots of the iTRAQ-labeled peptides were combined and cleaned with Pierce C18-spin columns (Thermo Scientific). The final peptide mixture was fractionated by reversed phase chromatography at basic pH using a C18 column (Zorbax Extend C18, 100 × 2.1 mm id, 3.5 μm, 300 Å, Agilent) mounted on the same HPLC system (HP1200, Agilent), at a flow rate of 0.2 ml/min. A total of 60 fractions were collected from each sample injection. The fractions were then combined according to their UV trace, desalted and loaded onto a reversed phase column C18 (Integrafit C18, Proteopep ™ II, 75 μm id, 10.2 cm, 5 μm, 300 Å, New Objective). This second separation was carry out at acid pH and at a constant flow rate of 350 nl/min. The microfractions were automatically spotted onto 1536 MALDI plates using a SunCollect MALDI Spotter (SunChrom). Finally, four plates containing 4 LC runs (corresponding to 4 samples) per plate were analyzed in a 4,800 MALDI-TOF/TOF instrument (ABSciex) with a Nd:YAG laser and a firing rate of 200 Hz. For MS full-scan acquisition, the instrument parameters were set as follows: m/z range from 800 to 4,000, fixed laser intensity of 3,500 kV and 1,500 shots/spectrum. For tandem MS acquisition mode, collision energy was set at 1 kV with CID gas (air) over a range from 60 to −20 m/z of the precursor mass value. A first MS/MS acquisition was performed with a fixed laser intensity of 4,300 kV and 2,000 shots/spectrum recorded. Up to 12 of the most intense precursors per spot with signal/noise ratio (S/N) > 80 were selected. An exclusion list was created to exclude from the analysis common contaminants such as trypsin autolysis peaks and matrix ion signals. Finally, a second MS/MS acquisition was performed excluding the precursors analyzed in the previous one and lowering the S/N threshold to 50 in order to detect those peptides that had not yet been fragmented.

### Analysis of the shotgun proteomics data

For protein identification and quantification, all the MS/MS data were analyzed using ProteinPilot™ software v.4.5 (Sciex). Each MS/MS spectrum was searched against the publicly available Uniprot/Swissprot database; the release version 2017_02 with 553,655 sequences, 198,177,566 residues and *Homo sapiens* taxonomy restriction was used. Trypsin cleavage specificity, methyl methanethiosulfate (MMTS) modified cysteine, biological modification “ID focus” settings and a protein minimum confidence score of 95% were set as fixed search parameters. The identity of the proteins was confirmed when the Detected Protein Threshold was >95% and the Unused ProtScore was >1.3. In order to relatively quantify the abundance of the proteins identified in each pool of samples, the ratios of the peak areas of iTRAQ reporter ions were calculated after data normalization to avoid loading error by bias and making the assumption that the labeled peptides were combined in 1:1 ratio. To remove background ion signal, peak areas of the iTRAQ reagents were also corrected by ticking the background correction option. Finally, we considered statistically significant only those changes with a *p* value ≤ 0.05, a ratio ≥1.3 (or ≤ 0.776) and an error factor (EF) < 2. For a more accurate data analysis, the Proteomics System Performance Evaluation Pipeline (PSEP), a tool integrated into the ProteinPilot software, was used to independently estimate the false discovery rate (FDR) using an automatic decoy database search strategy.

### Sample preparation for multiple reaction monitoring analysis with internal standards

The verification phase of this work was carried out using a targeted proteomic approach based on multiple reaction monitoring (MRM) analysis of 80 serum samples from the CHUS cohort. Firstly, protein concentration of each serum sample was determined by NanoDrop spectrophotometer (280 nm) and 10 μg of each sample were in-solution digested with trypsin following standard protocols. Briefly, proteins were denatured in 6M Urea/2M Thiourea/25 mM ammonium bicarbonate buffer and then reduced with 10 mM Dithiothreitol (DTT) for 1 h at 37°C. Then, cysteins were blocked by alkylation with 50 mM iodoacetamide (IA) for 45 min in the dark. Samples were diluted with 25 mM ammonium bicarbonate to lower the final urea concentration to 1 M and Promega Grade Trypsin was added at a 1:30 ratio (enzyme:protein). After 16 h at 37°C, the enzymatic digestion was stopped by lowering the pH of the reaction up to 2 with TFA. Finally, the digested peptides were cleaned-up by in-house made stage tips (3M Empore SPE-C18 disk, 47mm, Sigma Aldrich) after the addition of an experimentally determined amount of a mixture of stable isotope–labeled peptides (SIS, JPT, Germany). SIS peptides incorporated a fully atom labeled isotope at the C-terminal lysine (K) or arginine (R) position of each tryptic peptide, resulting in a mass shift of +8 for peptides ending in K (^13^C_6_,^15^N_2_) or +10 Da for peptides ending in R (^13^C_6_,^15^N_4_). Taking into account the different amounts and signals of the targeted peptides in serum samples, SIS peptides concentrations were individually determined and then adjusted to the level of their analogous endogenous peptides. Each sample protein digest was spiked with a constant amount of a SIS mixture ranging between 25, 50, 100 and 250 fmol/ul. Finally, samples were dried in a speed-vac concentrator with a refrigerated vapor trap.

### Liquid chromatography and multiple reaction monitoring (LC-MRM) analysis

Peptides mixtures composed of endogenous peptides (light peptides) and SIS peptides (heavy peptides) were analyzed by LC-MS/MS in a nanoLC system (TEMPO) coupled to a 5500-QTRAP instrument (Sciex). Peptides were desalted through a C18 column (5 μm particle size, 300Å pore size, 100 μm diameter and 2 cm length, Acclaim PepMap, Thermo Scientific) during 10 min at a flow rate of 3 μl/min and then separated on a C18 nanocolumn (75 μm internal diameter and 15 cm lenght, Acclaim PepMap 100, Thermo Scientific) at a constant flow rate of 300 nl/min. For the MRM method, we employed a chromatographic gradient which started with 5% of 0.1% Formic acid in 95% acetonitrile (buffer B). During the first 5 min the % of buffer B was increased up to 15%. Then, from min 5 until min 45, buffer B% increased up to 35% and, finally, it reached in one minute the maximum of acetonitrile concentration (95%B). After 10 mins in these conditions, B% was lowered down up to 5% in 1 min and finally the column was equilibrated in the same condition during 14 mins. Mass spectrometer was interfaced with a nano-spray source equipped with an uncoated fused silica emitter tip (20 μm inner diameter, 10 μm tip; New Objective) and operated in positive ion mode. The optimized conditions for the electrospray source were as follows: ion spray voltage, 2,600 V; curtain (CUR) gas, 20 psi; ion gas 1 (GS1), 25 psi; ion source gas 2 (GS2), 0 psi; collision activated dissociation (CAD) gas, medium and the interface heater temperature (IHT), 150°C. For both endogenous and SIS peptides, the optimal declustering potentials (DP) and collision energies (CE) were predicted employing the free software Skyline. The other compound dependent MRM parameters, the entrance potential (EP) and the cell exit potential (CXP), were experimentally set at 10 and 15, respectively. Q1 and Q3 were set to unit/unit resolution (0.7 Da) and pause between mass ranges set to 3 ms.

In order to avoid signal interference, we performed a screening based on the fragment-ion ratios of each transition taking into account the exported responses from the heavy and light peptides. To confirm that there was no signal at the same m/z transition values of the light peptides due to a possible contamination in the process of synthesis of the SIS peptides, we first analyzed the SIS mixture standard alone. Then, a digested pool of 4 RA sera (one serum sample from each group under study) was used as a background matrix. The pooled serum sample was analyzed without spiked heavy peptide standards, to prove that there were no hypothetically signal interferences at the m/z transition values of the heavy peptides. One ug of RA serum sample was injected on column. To test system reproducibility, two replicates were acquired for each sample. In order to avoid possible carry over in the nanoLC-MRM system, two blank injections were analyzed between each sample.

To ensure the correct identification of each peptide, we also performed a MRM Information Dependent Acquisition (IDA) experiment. When an individual MRM signal exceeded 1,000 counts, the mass spectrometer automatically switched from MRM to EPI scanning mode. Each precursor was fragmented a maximum of twice before and then excluded for 10 s; the masses were scanned from 250 to 1,000 Da. The rolling collision energy (CE) option was employed to automatically ramp up the CE value in the collision cell as the m/z values increased.

### Analysis of the MRM data

Skyline 1.3 software (MacCoss, Seattle, WA, USA) was used for MRM method refinement and optimization, peak integration and quantitative analysis of MS data generated from targeted experiments. The selection of peptides for the verification analysis was based on the intensity order of transitions, the relative retention times (RT) across runs, and the co-elution of endogenous light peptide (L) with the stable isotope-labeled standard (SIS) reference heavy peptide (H) spiked in the sample. Once the raw data files were loaded into Skyline, they were manually inspected to ensure correct peak detection and accurate integration. The Savitzky-Golay filter were applied for the purpose of smoothing the data. Peak area ratios between light and heavy peptide were used to calculate the relative peak area ratio of each peptide object of the study. Finally, customized data reports were generated and exported to Excel for further analysis. Mean, standard deviation and coefficient of variation (% CV) of the mean peak ratio area and retention time (RT) were calculated for all RA sample replicates.

### ELISA set up and analysis

ELISA development kits were purchased from Bio-Techne (Minneapolis, USA, DuoSets DY3694 and DY8465). The full-matching of the immunogen sequence of the antibodies with the peptide fragments analyzed by MRM was a requirement for kit selection. Additionally, TMB Substrate Solution was acquired from ThermoFisher (Massachusetts, USA). H_2_SO_4_ 1N was used as Stop Solution. Optical density measurements were assessed at 450 nm, using the Infinite M200 Nanoquant plate reader (Tecan, Switzerland). Wavelength correction for optical imperfections in the plate was set at 550 nm, as readings made directly at 450 nm without correction may be higher and less accurate. Sample dilution tests were performed as detailed in each specific ELISA kit, using samples from each condition. Sample concentrations were calculated based on each plate's calibration curve with the GraphPad Prism software.

### Sandwich immunoassay on suspension bead arrays

RET4 levels were measured by a bead-based sandwich immunoassay (Duo set, R&D Systems, Minneapolis, MN, USA) that was previously developed and analytically validated in our group as part of a multiplex panel (20). Antibodies and recombinant protein were prepared following manufacture instructions and the suspension bead array was created as previously described (21). In short, the capture antibody for RET4 was coupled to carboxylated color-coded magnetic beads using 10 mg/ml of ethyl-3-(3-dimethylaminopropyl) carbodiimide (EDC) (03449, Sigma-Aldrich) and 10 mg/ml of sulfo-N-hydroxysulfosuccinimide (Sulfo-NHS) (24510, Thermo-Fisher Scientific) in phosphate buffer. Serum samples were diluted 1/10,000 in PBS-T 0.05%. A seven-point standard curve covering a range of 80–0.10 μg/ml was prepared by using three-fold serial dilutions in assay buffer (1% BSA in PBST 0.05%). Assay buffer was used as a background sample. For assay performance, 25 ul of the diluted samples, standards, and background sample were added to appropriate wells containing 1,000 coupled beads and incubated at room temperature for 2 h with gentle agitation. After incubation, the beads were washed three times with PBS-T 0.05%. Subsequently, 25 μl of biotinylated detection antibody was added at 1 μg/ml in PBS-T 0.05% and incubated for 1 h at room temperature with shaking. The beads were then washed again before incubation with 25 μl of a 1/500 streptavidin-phycoerytherin (SAPE) (Thermo) for 20 min at room temperature in shaking. The beads were finally washed, suspended in 100 μl of PBS-T 0.05% and analyzed on a MagPix instrument (Luminex corp.). GraphPad Prism software was used to generate a five-parameter logistic (5-PL) curve-fit and interpolate the RET4 concentration contained in the sample. The final sample concentration was obtained taking into account the dilution factor employed.

### Statistical analysis

For the analysis of shotgun proteomics data, the statistical package from ProteinPilot were employed. The results obtained after applying the proper normalization tools were exported to Microsoft Excel for further analyses. Where appropriate, results were expressed as the mean ± standard error (Tables 1–3). GraphPad Prism 7 was used for the statistical analyses of the data from verification (MRM) and validation (ELISA) phases. Kruskal–Wallis test multiple comparison with *post-hoc* correction was applied to compare means among the four different groups of patients. A *p* < 0.05 was considered statistically significant.

**Table 2:** Results from the iTRAQ-LC-MS/MS analysis of the discovery phase.

**Accession**	**Symbol**	**Name**	**Peptides**	**Group**	**RF**+**ACPA**+		**RF**+**ACPA–**		**RF-ACPA**+	
					**Mean ratio**	**SD**	**Mean ratio**	**SD**	**Mean ratio**	**SD**
P43652	AFAM	Afamin	36	+–			1.30	0.03		
P02763	A1AG1	Alpha-1-acid glycoprotein 1	18	++/+–	11.2	1.89	4.27	0.60		
P19652	A1AG2	Alpha-1-acid glycoprotein 2	11	++	6.34	0.71				
P01011	AACT	Alpha-1-antichymotrypsin	50	++/–+	1.74	0.17			1.81	0.24
P01009	A1AT	Alpha-1-antitrypsin	11	+-/–+			1.71	0.16	0.33	0.07
P02765	FETUA	Alpha-2-HS-glycoprotein	44	+–			1.56	0.16		
P02647	APOA1	Apolipoprotein A-I	6	+–			2.45	0.38		
P02652	APOA2	Apolipoprotein A-II	5	–+					0.49	0.04
P04114	APOB	Apolipoprotein B-100	180	++/–+	0.18	0.02			1.45	0.14
P02649	APOE	Apolipoprotein E	11	+–			1.92	0.07		
P02749	APOH	Beta-2-glycoprotein 1	45	+–			1.86	0.16		
P00450	CERU	Ceruloplasmin	88	++	1.41	0.08				
P01024	CO3	Complement C3	51	+–/–+			0.29	0.07	0.58	0.04
P00738	HPT	Haptoglobin	15	++/+–/–+	2.79	0.20	0.74	0.08	0.38	0.03
P19827	ITIH1	Inter-alpha-trypsin inhibitor heavy chain H1	39	–+					1.41	0.07
P19823	ITIH2	Inter-alpha-trypsin inhibitor heavy chain H2	39	+–			0.74	0.03		
P01042	KNG1	Kininogen-1	39	+–			1.40	0.06		
P05155	IC1	Plasma protease C1 inhibitor	26	++/–+	1.56	0.21			1.59	0.24
P00747	PLMN	Plasminogen	18	++/–+	1.66	0.04			0.66	0.03
P00734	THRB	Prothrombin	72	+–			1.57	0.05		
P02753	RET4	Retinol-binding protein 4	25	++	1.69	0.04				
P02766	TTHY	Transthyretin	4	++/+–	0.23	0.04	2.00	0.20		
P02774	VTDB	Vitamin D-binding protein	112	++/+–/–+	1.54	0.26	3.05	0.47	2.15	0.29
P04004	VTNC	Vitronectin	31	+–			1.61	0.16		
P25311	ZA2G	Zinc-alpha-2-glycoprotein	42	+–			1.82	0.30		

++: RF+ACPA+; +–: RF+ACPA–; and –+: RF–ACPA+. Ratios were calculated in comparison to the double negative (RF–ACPA–). Only proteins with p < 0.05 in the comparisons are included. Details on the data obtained from the independent pools are shown in Supplementary Table S2.

**Table 3:** Peptides showing differential abundance between groups in the MRM quantitative targeted analysis of the verification phase.

**Accession**	**Symbol**	**Peptide**	**RF–/ACPA–**		**RF**+**/ACPA**+		**RF**+**/ACPA–**		**RF–/ACPA**+	
			**Mean L/H ratio**	**SD**	**Mean L/H ratio**	**SD**	**Mean L/H ratio**	**SD**	**Mean L/H ratio**	**SD**
P04114	APOB	ATFQTPDFIVPLTDLR	0.45	0.28	0.47	0.21	0.40	0.12	0.49	0.26
P04114	APOB	IPSVQINFK	1.37	0.66	1.65	0.67	1.59	0.53	1.63	0.81
P04114	APOB	TSSFALNLPTLPEVK	0.35	0.26	0.32	0.10	0.34	0.09	0.33	0.11
P05155	IC1	GVTSVSQIFHSPDLAIR	0.56	0.32	0.65	0.18	0.57	0.19	0.57	0.31
P05155	IC1	LEDMEQALSPSVFK	0.35	0.17	0.43	0.13	0.42	0.20	0.42	0.37
P00747	PLMN	WELCDIPR	0.75	0.19	0.81	0.18	1.20	0.31	1.24	0.47
P00747	PLMN	VIPACLPSPNYVVADR	0.15	0.05	0.16	0.03	0.18	0.04	0.17	0.05
P00747	PLMN	EAQLPVIENK	0.32	0.08	0.33	0.07	0.36	0.07	0.39	0.12
P02753	RET4	YWGVASFLQK	0.39	0.23	0.41	0.14	0.45	0.12	0.42	0.15
P02753	RET4	LLNLDGTCADSYSFVFSR	1.81	1.60	1.68	0.86	0.96	0.28	1.00	0.42
P02774	VTDB	YTFELSR	0.68	0.21	0.77	0.19	0.80	0.21	0.82	0.30
P02774	VTDB	HLSLLTTLSNR	0.67	0.22	0.75	0.20	0.76	0.18	0.83	0.22
P02774	VTDB	VLEPTLK	0.61	0.14	0.67	0.17	0.68	0.16	0.77	0.25
P02763	A1AG1	YVGGQEHFAHLLILR	0.99	0.41	1.44	0.55	1.21	0.50	1.20	0.49
P02763	A1AG1	EQLGEFYEALDCLR	1.21	0.72	1.58	0.73	1.13	0.49	1.13	0.66
P02763	A1AG1	SDVVYTDWK	2.00	0.81	3.01	1.14	3.24	1.33	3.64	2.82
P19652	A1AG2	EHVAHLLFLR	0.52	0.14	0.60	0.19	0.58	0.16	0.65	0.23
P19652	A1AG2	EQLGEFYEALDCLCIPR	8.91	6.76	10.71	6.00	7.02	2.35	6.63	2.80
P19652	A1AG2	SDVMYTDWK	1.02	0.34	1.19	0.65	1.20	0.44	1.41	0.68
P01011	AACT	EIGELYLPK	0.21	0.07	0.30	0.12	0.28	0.13	0.30	0.15
P01011	AACT	AVLDVFEEGTEASAATAVK	5.15	2.42	6.82	3.36	6.19	2.82	5.93	2.66
P00738	HPT	VGYVSGWGR	1.86	0.74	2.50	0.75	2.30	1.11	2.27	1.19
P00738	HPT	VTSIQDWVQK	2.01	0.88	2.74	0.91	2.32	1.06	2.30	1.19
P43652	AFM	ESLLNHFLYEVAR	0.29	0.24	0.25	0.10	0.22	0.05	0.23	0.06
P43652	AFM	DADPDTFFAK	0.44	0.12	0.45	0.13	0.52	0.12	0.64	0.19
P43652	AFM	FTFEYSR	0.63	0.27	0.65	0.22	0.68	0.14	0.75	0.17
P02765	AHSG	EHAVEGDCDFQLLK	1.86	0.53	1.91	0.45	2.06	0.57	1.85	0.44
P02765	AHSG	FSVVYAK	0.43	0.13	0.44	0.12	0.48	0.13	0.48	0.13
P01042	KNG1	YNSQNQSNNQFVLYR	4.22	1.39	4.64	1.20	5.01	1.05	5.34	1.80
P01042	KNG1	TVGSDTFYSFK	0.49	0.15	0.52	0.08	0.56	0.12	0.61	0.20
P01042	KNG1	YFIDFVAR	0.65	0.36	0.67	0.19	0.73	0.15	0.69	0.17

Data are expressed as the mean L/H ratios calculated by MRM analysis. Quantification data from each individual sample (n = 80, by duplicate) are shown in Supplementary Table S4. SD, standard deviation.

## Results

### Discovery phase screening by iTRAQ-LC-MS/MS analysis

**Figure 2:**
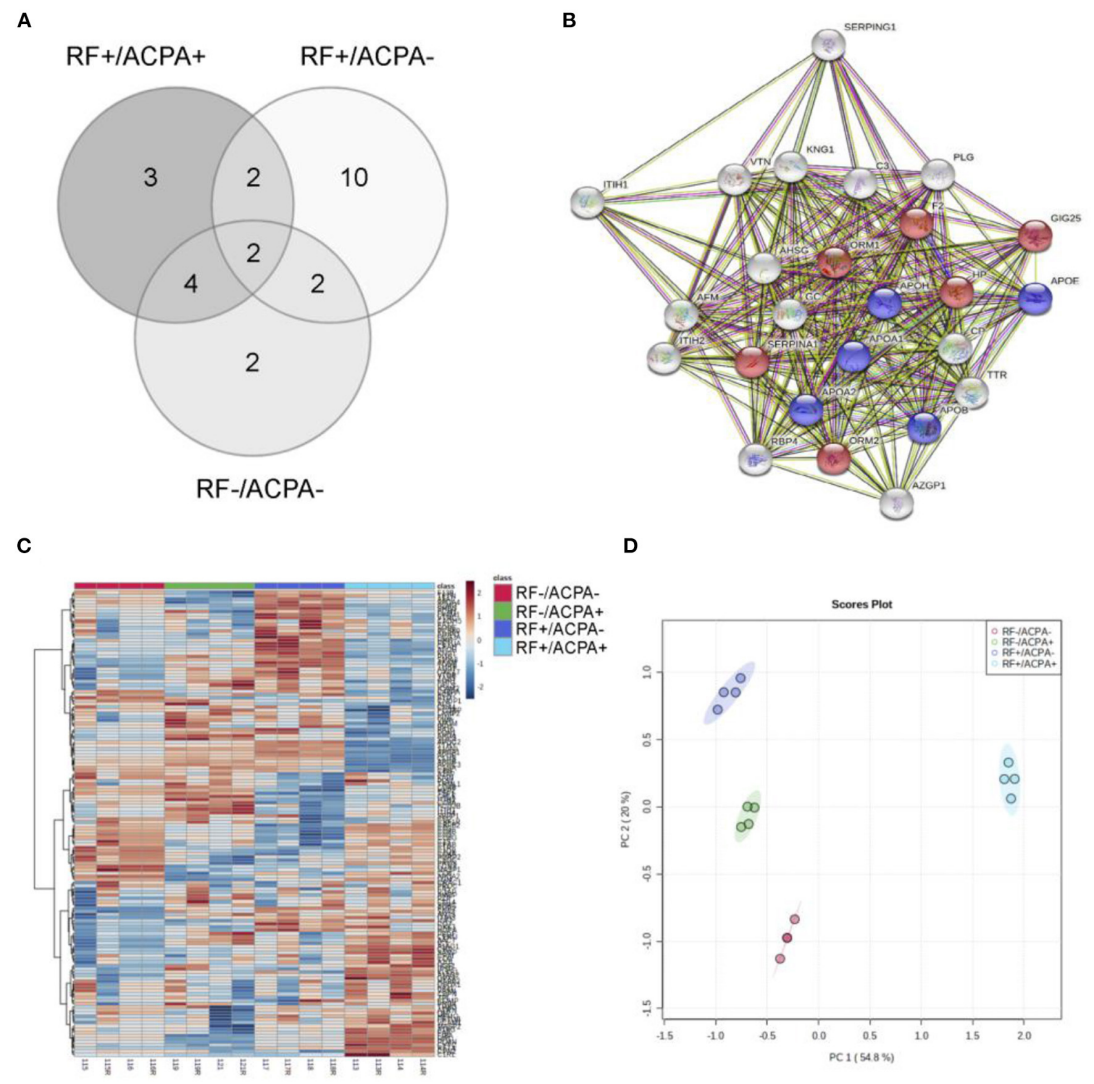
Results from the discovery phase. **(A)** Overlapping of the 25 differential proteins observed between the four groups under study (+/+, +/–, –/+ and –/–). **(B)** Pathway analysis obtained with STRING software, showing the involvement of most differential proteins in inflammatory (red) and lipid metabolism (blue) processes. Grey dots represent involvement in other not significantly enriched pathways. **(C)** Hierarchical clustering showing the differential protein profiles observed in the sera from the four groups. **(D)** Principal component analysis illustrates the perfect separation of the groups based on their proteomic profiles.

For the initial screening, 80 sera from the CHUS cohort were grouped into 4 pools (20 patients/pool) according to their ACPA/RF status and then analyzed by nanoLC-MALDI/MS. Overall the number of proteins identified with at least 1 peptide was 153 and they are listed in Supplementary Table S1. Using an iTRAQ-8plex technology-based quantitative proteomic approach, we were able to quantify all of them except one. In order to ensure maximum robustness of the quantitative results we only considered those proteins identified with two or more peptides. From the 126 proteins that met this criterion, 25 showed a statistically significant modulation in at least one group compared with the double negative group (Table 2). The overlapping of this significant alteration between groups is shown in Figure 2A. A functional pathway analysis revealed that the modulated proteins were related mainly to inflammatory processes (acute phase reactants) and lipid metabolism (Figure 2B). As shown in Figure 2C, heatmap clustering analysis showed the differentially expressed proteins in the four groups. Finally, an unsupervised Principal component analysis (PCA) was performed using the quantification data of each pool. As shown in Figure 2D, RA patients were perfectly classified into the four groups according to their serological status. The quantitative results obtained in this shotgun analysis are detailed in the Supplementary Table S2. Among all quantified proteins, the abundance of 11 was altered in the sera from RF+/ACPA+ patients, 16 in RF+/ACPA– patients and 10 in RF–/ACPA+ patients compared to RF–/ACPA– ones. Three proteins were exclusively modulated between double seropositive and double seronegative group (AIAG2, CERU and RET4), being all of them increased in double seropositive patients. Ten proteins were found uniquely altered in the RF+/ACPA– group (AFAM, FETUA, APOA1, APOE, APOH, ITIH2, KNG1, THRB, VTNC, ZA2G) whereas APOA2 and ITIH1 were found altered only in the RF–/ACPA+ condition. Finally, haptoglobin (HPT) and vitamin D binding protein (VTDB) were the unique proteins modulated in the sera of patients belonging to all the seropositive groups (RF+ACPA+, RF–ACPA+ and RF+ACPA–), when compared to the double seronegative one. All these proteins were selected for further verification in individual samples by targeted mass spectrometry (MRM).

### Verification phase by targeted MRM quantification

A multiplex MRM assay was then developed to verify the differential abundance of the set of biomarker candidates identified in the shotgun screening. After an optimization process, we were able to detect and quantify simultaneously 12 of the 25 protein biomarker candidates that could be associated with RF and/or ACPA. Thirty-one peptides belonging to these 12 target proteins were selected and analyzed using the Skyline software. The Supplementary Table S3 shows the list of the final transitions (*n* = 180) and the settings employed for their analysis. For this biomarker verification phase, all the samples from the CHUS cohort were analyzed individually (*n* = 80) and measured in duplicate (*n* = 160). All the data collected in this analysis are detailed in Supplementary Table S4, including the retention times (RT), mean RT, peak areas ratios, mean peak area ratios and CVs obtained for each peptide in each sample. The results from the relative quantitation of the proteins between the different groups that were compared (RF+/ACPA+, RF+/ACPA–, RF–/ACPA+, RF–/ACPA–) are presented as average values of the peak areas out of all transitions and peptides per protein, after an intensity normalization step with their corresponding heavy isotope–labeled standard references (L/H mean peak ratio).

**Figure 3:**
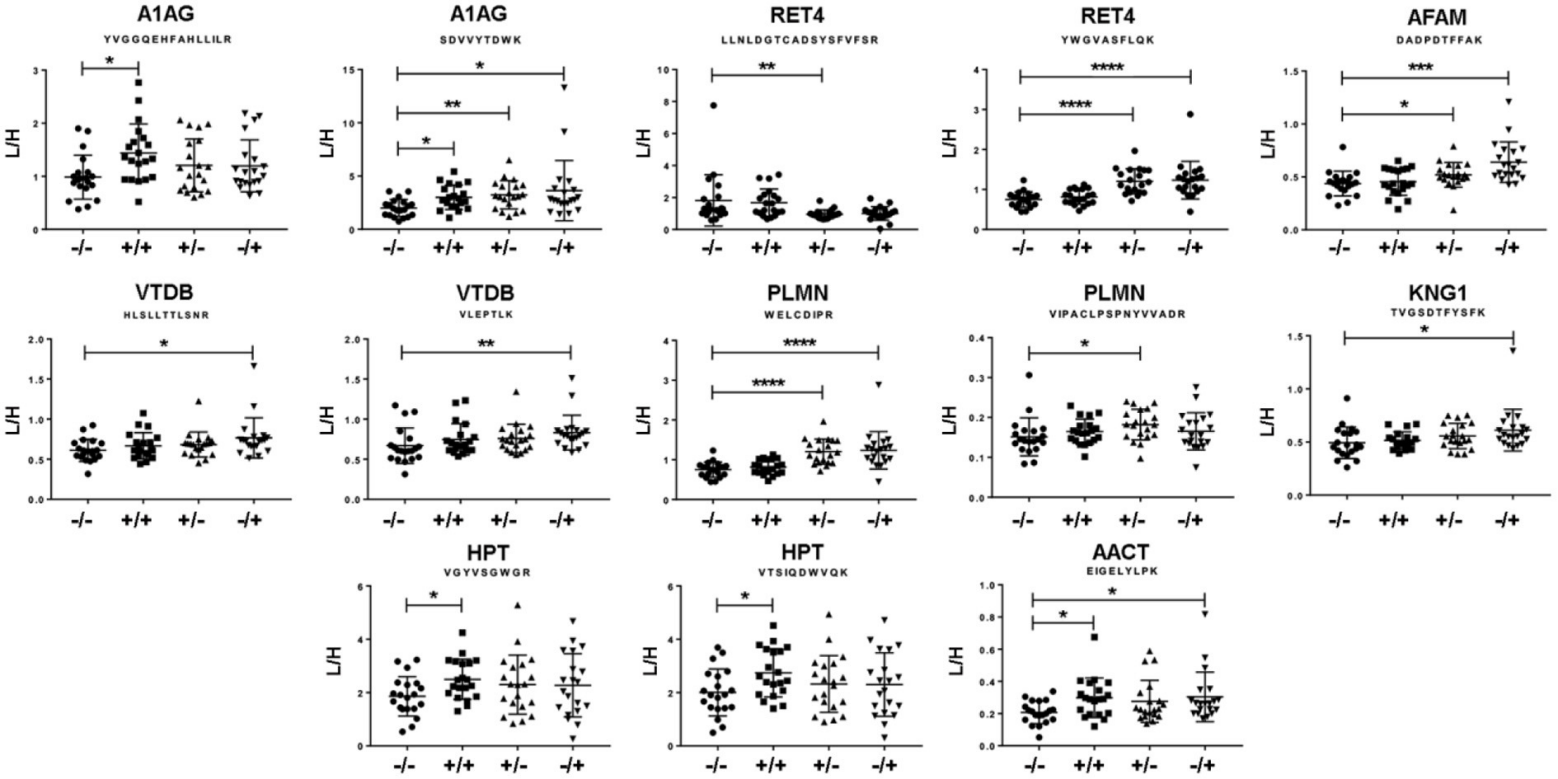
Results from the verification phase. Peptides showing differential abundance between groups, as observed by MRM analysis on individual samples from the cohort of Santiago de Compostela. Data are expressed as heavy/light mean peak ratios (L/H). * *p* < 0.05; ** *p* < 0.01; *** *p* < 0.005 and **** *p* < 0.001.

A significant modulation of 13 peptides belonging to 8 different proteins was observed (Figure 3). All of them were decreased in the double seronegative group (our reference group) except one peptide belonging to RET4. Five peptides (two from A1AG1, two from HPT and one from AACT) were increased in the double seropositive group, being three of them specific of this condition. Five peptides (two from RET4 and one from A1AG1, AFAM, and PLMN) were found altered in RF+/ACPA– group, being one of RET4 decreased in this condition. Finally, 8 peptides belonging to 7 proteins (4 were the same as the RF+/ACPA– group plus AACT, KNG1 and VTDB) were found altered in RF–/ACPA+, always in comparison with the RF–/ACPA– reference group (Table 3). Three acute phase reactants (A1AG1, HPT and AACT) displayed the same modulation in both screening and verification phases, thus confirming their association with the double positivity status. Two of them were selected for the next validation step along with RET4. The rationale for selecting RET4 was based on the increment observed for one of its peptides (LLNLDGTCADSYSFVFSR) in the double seronegative patients compared to the RF+/ACPA– group.

### Validation phase by immunoassays

**Figure 4:**
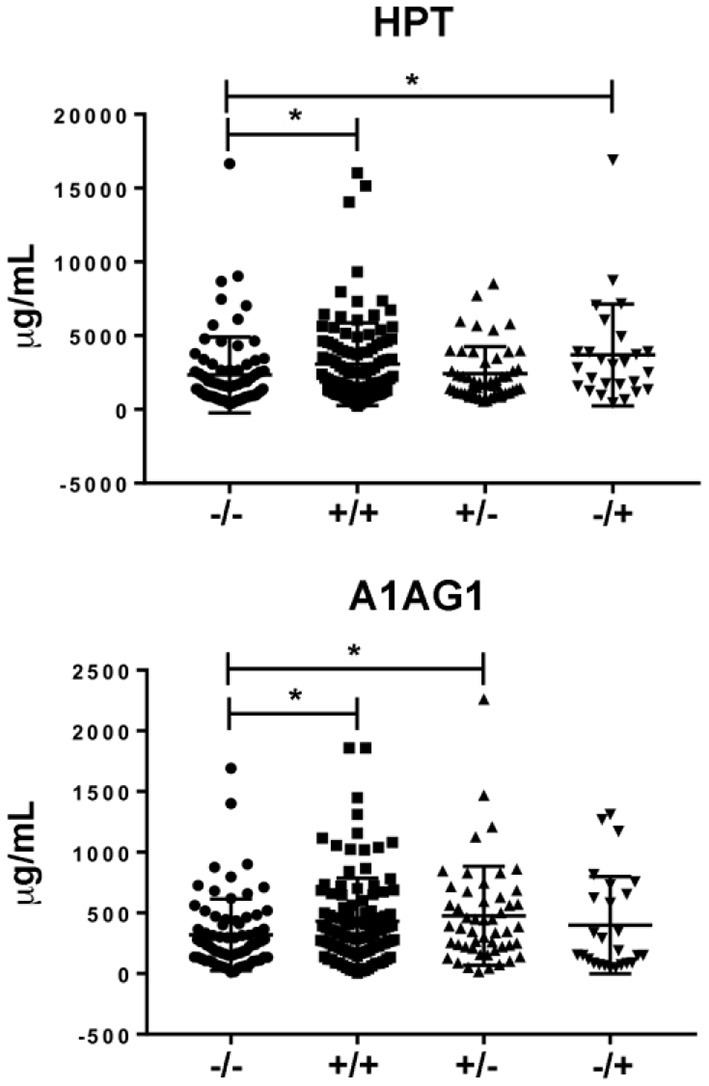
Results from the validation phase. Different levels of HPT [at the **(top)**] were found between the double positive group and the FR–/ACPA+ group compared to the double seronegative. In the case of A1AG1 **(bottom)** the difference was found between the two groups with FR+ compared to the double seronegative. * *p* value ≤ 0.05.

Taking into account the results obtained in the verification phase, we performed an orthogonal validation by immunoassays in the whole cohort from CHUAC (*n* = 260). Serum levels of A1AG1 and HPT were measured by conventional ELISA immunoassays, whereas RET4 was quantified in the same cohort using suspension beads arrays. The quantitative data obtained in this validation step are reported in the Supplementary Table S5. As shown in Figure 4, the results from this analysis confirmed the elevated values of A1AG1 and HPT in the double seropositive patients (*p* = 0.009 A1AG1; *p* = 0.003 HPT). The increased level of A1AG1 was found associated with the RF rather than the ACPA status (*p* = 0.023 RF+/ACPA–), whereas HPT was associated with ACPA rather than RF (*p* = 0.013 RF–/ACPA+). On the other hand, data obtained for RET4 did not confirm the MRM results, as no significant differences were detected among the four studied groups.

### Discussion

Autoantibody-negative and autoantibody-positive RA are considered two entities with different underlying pathophysiological mechanisms, long-term outcomes and disease presentations (22). However, the effect of the presence of RF and ACPA autoantibodies on clinical phenotypes remains unclear. Despite their routine use in clinical practice as biomarkers for the diagnosis of RA, their specificity and sensitivity are quite low (especially in the case of RF). Furthermore, these autoantibodies are often discordant with each other and up to 30% of RA patients never develop them. Our goal in this work has been to describe the molecular endotypes associated with the serological phenotype of the RA patients in order to improve diagnostic tools currently available. Identifying specific RA phenotypes and endotypes can inform about disease prognosis and guide therapeutic development with the potential of increasing personalized strategies and positively impacting patient care. Currently, the most common method for subgrouping RA patients is based primarily on clinical phenotypes. However, the need to identify patient endotypes to targeted treatments has gained increasing importance, particularly from the point of view of new drug discovery, for which identifying the correct target is key to success (17).

In this study, a classical biomarker pipeline based on quantitative proteomics has been followed for the discovery, verification and validation of putative serum protein biomarkers that could improve the characterization of RA endotypes (23). As stated previously (24), this pipeline is based on a reverse interrelationship among the number of samples used for the analysis and the number of proteins that arise as possible biomarkers. In most previous proteomics studies, blood samples were pooled for MS analysis (25). This strategy has been extensively reported as a valid and valuable procedure when applied to the biomarkers discovery phase (26), although consideration should we taken when interpreting the results since it may lead to loss of information. Therefore, we decided to follow this pooling approach in the discovery phase but then move to individual targeted MS analysis for the verification phase. The principal bottleneck of the biomarker pipeline is the lack of verification step as a bridge between discovery and validation phases (27). In order to overcome these limitations, we present a stepwise workflow to identify RA biomarkers and verify them by targeted MS-based analysis before their validation by immunoassays. In this work, the importance of a robust verification phase to fill this gap clearly emerges and the benefits of targeted mass spectrometry for this purpose are also highlighted. We chose LC/MRM-MS as the ideal technique for the protein quantification of our clinical samples mainly due to its multiplexing potential (31 peptides from 12 proteins, in our case), high specificity (each target peptide is unique and does not interfere with other peptides), high sensitivity (limit of quantification in the range of pg/ml), small amount of sample (2 ul crude serum) and no need for antibodies or other means of sample depletion or enrichment (25). These characteristics have turned MRM-MS the reference method for the accurate quantification of peptides in biological matrices (28). In this study, the development of a robust MRM method allowed us to classify RA patients according to their serology.

The conventional methods for distinguishing between clinical phenotypes and identifying specific therapeutic targets might not be sufficient in complex diseases such as RA. Novel approaches are therefore of great interest. The knowledge on molecular endotypes can be unraveled with sensitive techniques such as high-throughput proteomics. This approach may be used to stratify specific subgroups of RA patients that could benefit from a variety of targeted preventive and treatment strategies that ultimately facilitate the development of personalized medicine strategies. In the proteomic field, technological advances born under the Human Proteome Project have been extremely valuable (29). Thousands of proteins can be analyzed in plasma or serum by mass spectrometry-based proteomics technologies. However, despite these important accomplishments, the proteome complexity of serum samples still exceeds the technical capability of the MS instruments. For this reason, we carried out an immunodepletion step in the discovery phase, in order to enrich the RA serum samples of low abundant proteins by reducing their dynamic range and thus achieving greater sensitivity. The LC-MS workflow employed gave us a robust tool to dig deeper into the complex biology of the RA cellular machinery and the potential to discover low-level, biologically significant proteins, and validate them in our translational and clinical proteomics research. Pathway enrichment analysis highlighted the top enriched pathways in RA (see Figure 2): inflammation and lipid metabolism. These two major pathways are strictly interconnected, as lipids are involved in several biological processes. Apart of being the main component of cell membranes, they regulate cell migration, immune cell plasticity and inflammation. Our results are in agreement with those from Luan et al. (30), supporting their role in perpetuating the inflammatory state characteristic of RA patients.

Furthermore, the protein biomarkers detected in our study through serum protein profiling embody other physiological changes in RA apart from those in expected pathways such as inflammation and immunity. Functional analysis of the results from the discovery phase (shotgun proteomics) revealed that GO processes with the lowest *p*-values were platelet degranulation, acute inflammatory response, transport, acute-phase response and negative regulation of blood (Supplementary Table S6). Cholesterol metabolism and complement and coagulation cascades were the two KEGG pathways significantly associated with the 25 proteins differentially expressed among the four groups. Seven of the altered proteins are inflammatory markers also known as acute phase reactants (APRs). APRs are key regulators of the immune response. They function as mediators and/or inhibitors of inflammation and act as carrier proteins for the products generated during the inflammatory process. In addition, they can also play an active role in tissue repair and remodeling. Interestingly, these proteins have been extensively defined as acute phase and/or disease activity markers in several inflammatory conditions including RA (31, 32), although they have not yet been established as a multi-biomarker panel for clinical utility. In fact, the only multi-biomarker disease activity test for RA (33) commercially available does not include any of these proteins.

Analysis of individual serum samples in the verification phase revealed differentially expressed proteins in the four groups, among which A1AG1, RET4, AFAM, PLMN, KNG1, HPT, AACT and VDBP emerged as novel stratification biomarkers by MRM quantification. Considering the data from this targeted analysis, the GO term 0051180 (vitamin transport) appeared among the most significant processes in the network function analysis along with the previous ones (Supplementary Figure S2), thus confirming the alterations observed in the discovery phase. RET4, AFAM and VDBP belong to this functional group. RET4 is involved in the vitamin A transport regulation through blood plasma from the deposit located into the liver to peripheral tissues. This vitamin is essential for different cellular processes such as cell growth, cell differentiation and bone development, thus its deregulation could influence bone regeneration in patients suffering from erosion due to RA (34). Recent studies suggested that elevated serum levels of RET4 were associated with increased risk of insulin resistance in newly diagnosed and untreated RA patients (35). In addition, blood coagulation disease was significantly associated with proteins found altered in the targeted analysis. Our results are in accordance with previous publications demonstrating the role of blood coagulation in the pathophysiology of RA (25, 36, 37). Likewise, in a previous study, abnormally activated coagulation was reported due to the altered expression of coagulation-related factors in patients with RA and aggravated RA (38). Overexpression of serum fibrinogen, increased platelets and plasmin activity were associated with RA. In that study, functional analysis showed that serum proteins from RA patients were maximally associated with blood coagulation. Furthermore, in both pathway maps and network functional analyses, the complement system was activated in RA patients compared to the control group. Complement activation is triggered by three major pathways (lectin-induced, classical and alternative) that are mediated by differentially expressed serum proteins and membrane-associated proteins (39, 40). In our work, we confirmed that the differential proteins identified in RA patients were involved in all of them. Our results are in line with others that had previously shown that the complement pathway is clearly activated in RA patients (41, 42).

Finally, in the validation phase, we confirmed by immunoassays the MRM results for two of these proteins: HPT and A1AG1. As reported in results section, MRM findings indicate that two specific C-terminal HPT fragments (VGYVSGWGR and VTSIQDWVQK) and two specific A1AG1 fragments (YVGGQEHFAHLLILR and SDVVYTDWK) might be applied as novel biomarkers for the diagnosis and prognosis of RA. The individual differences in their expression, dependent on the serological status of RA patients, were confirmed in our validation cohort of 260 RA patients. In the CHUAC cohort, we found that individuals with the seropositive phenotype had relatively higher levels of HPT and A1AG1 in circulation compared with those with the seronegative phenotype. The HPT increase seems to be specifically associated to the ACPA positive status, whereas the A1AG1 increase seems to be specifically associated to the RF positive status more than to the ACPA positive status (Supplementary Figure S3). Therefore, our work provides novel information for the classification of the seropositive phenotype in RA, with the characterization of two new clusters of patients: one ACPA+HPT+ and the other RF+A1AG+. This is the first time these proteins emerge as directly related with the serological phenotype of RA patients. Altogether, their measurement could aid RA classification and provide a novel molecular portrait of the patient, thus representing an additional tool for precision medicine strategies. A further exploration of the associations of these endophenotypes with other previously studied, such as erosiveness or response to methotrexate, would add valuable information to advance in this area.

## Study limitations

The impossibility of better characterizing the population object of this study (CHUS and CHUAC cohorts) limits the interpretation of the results that have been obtained. In particular, we have not been able to gather information about the treatment of the patient, the duration of the disease at presentation, disease activity or possible extra-articular manifestations at the time of sample extraction. Nevertheless, although this lack of information has hindered the development of more extensive statistical analyses and models for RA endophenotyping, the characterization of the two novel clusters of patients that we describe in this study undoubtedly mean a progress in the classification of this disease at the molecular level. Further studies on well-characterized cohorts are needed to confirm this aspect, and also to move forward in the application of the results obtained herein into precision medicine tools.

## Conclusions

Taking into account the results obtained in the different phases of the study (Figure 1), from the discovery to the validation phase, we show that HPT and A1AG1 may be complementary to RF and ACPA to improve their sensitivity and/or specificity. The clinical utility of the protein profiles reported herein remains to be tested. Further efforts should be invested to facilitate progress in the development of biomarkers for RA diagnosis and stratification. Identification of subgroups of arthritis patients would present a significant advance in selecting the most effective treatment for an individual patient (43). The personalized treatment, informed by biomarkers, is only feasible by increasing knowledge on the molecular profiles underlying the phenotypes: disease signatures and patient endotyping. The characterization of specific endotypes associated with the serological status of the patients, carried out in this study, provides additional information that may be useful to move forward in precision medicine strategies.

## Data availability statement

Data are available via ProteomeXchange with identifier PXD037386.

## Ethics statement

The studies involving human participants were reviewed and approved by Comité de Ética de la Investigación de A Coruña—Ferrol, Galicia, Spain. The patients/participants provided their written informed consent to participate in this study.

## Author contributions

VC and FB conceived and designed the study. EP-P and AG provided samples for the discovery and verification phases of the study. VC, PF-P, and CR-R developed and validated the MS-based experiments with support from LG and AI. VC and LL developed and validated the immunoassays with support from RP and PQ. VC, PF-P, and CR-R performed the statistical analysis. VC, CR-R, PF-P, and FB interpreted the data and drafted the initial manuscript. All authors have made significant contributions to the conception and design of this study, the acquisition of data, its analysis and interpretation, contributed to revision and editing of the manuscript and approved the version to be submitted, and were involved in drafting the article and approved the final version to be published.

## Funding

This study has been funded by Instituto de Salud Carlos III through the projects PI16/02124, PI17/00404, PI19/01206, PI20/00793, RETIC-RIER RD16/0012/0002, and RICORS-REI RD21/0002/0009 (co-funded by European Regional Development Fund/European Social Fund; A way to make Europe/Investing in your future). This study was also supported by AE CICA-INIBIC (ED431E 2018/03) and grants IN607A2021/07 and IN607D2020/10 from Xunta de Galicia. The Biomedical Research Networking Center (CIBER) is an initiative from Instituto de Salud Carlos III (ISCIII). PF-P was supported by Contrato PTA (PTA2017-14846-I), Ministerio de Ciencia, Innovación y Universidades. LL was supported by Contrato Sara Borrell (CD19/00229), Fondo de Investigación Sanitaria, ISCIII. VC is supported by RETIC-RIER RD16/0012/0002 and RICORS-REI RD21/0002/0009. Funding for open access charge: Universidade da Coruña/CISUG.

## Conflict of interest

The authors declare that the research was conducted in the absence of any commercial or financial relationships that could be construed as a potential conflict of interest.

## Publisher's note

All claims expressed in this article are solely those of the authors and do not necessarily represent those of their affiliated organizations, or those of the publisher, the editors and the reviewers. Any product that may be evaluated in this article, or claim that may be made by its manufacturer, is not guaranteed or endorsed by the publisher.

## Abbreviations

A1AG1: Alpha-1-acid glycoprotein 1
AACT: Alpha-1-Antichymotrypsin
ACPA: Anti-citrullinated protein antibodies
AFAM: Afamin
CHUAC: Complejo Hospitalario Universitario de A Coruña
CHUS: Complejo Hospitalario Universitario de Santiago de Compostela
CV: Coefficient of Variation
GO: Gene Ontology
HPT: Haptoglobin
iTRAQ: Isobaric tag for relative and absolute quantitation
KNG1: Kininogen-1
LC: Liquid Chromatography
MALDI-TOF: Matrix-Assisted Laser Desorption/Ionization—Time-Of-Flight
MRM: Multiple Reaction Monitoring
MS: Mass Spectrometry
PLMN: Plasminogen
RA: Rheumatoid Arthritis
RET4: Retinol 4 Binding Protein
RF: Rheumatoid Factor
RT: Retention Time
VDBP: Vitamin D Binding Protein.
