# Supplementary material for: Association of the serological status of rheumatoid arthritis patients with two circulating protein biomarkers: A useful tool for precision medicine strategies
Source: Front Med (Lausanne). 2022 Oct 28;9:963540. doi: 10.3389/fmed.2022.963540 (PMC9651940; doi:10.3389/fmed.2022.963540)
Supplement: Supplementary file 1 [file Data_Sheet_1.docx]

Supplementary Material

# Supplementary Tables

**Supplementary Table S1.** MRM Transitions analyzed in this work and settings for their analysis.

**Supplementary Table S2.** List of serum proteins identified in the shotgun LC-MS/MS analysis of the discovery phase.

**Supplementary Table S3.** Quantification data obtained for the proteins significantly modulated between the different groups in the discovery phase.

**Supplementary Table S4.** Results from the MRM analysis of the verification phase. A) Mean, standard deviation and coefficient of variation (CV) for the peak area ratio of the light (or endogenous) and the heavy peptide. B) Retention time (RT) for each sample analyzed in the MRM method.

**Supplementary Table S5.** Results from the validation phase obtained by immunoassay analysis on a cohort from A Coruña.

**Supplementary Table S6**. GO analysis of the differential proteins identified in this work in A) the discovery phase (n=25) and B) the verification phase (n=12).

# Supplementary Figures


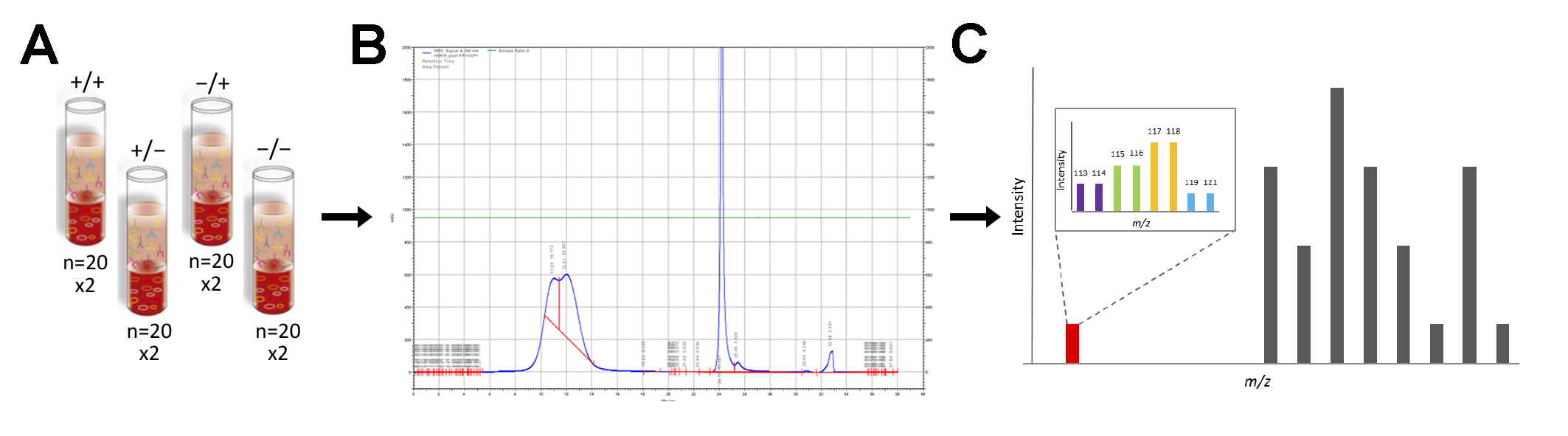


**Supplementary Figure S1.** Workflow followed in the discovery phase of this work. A) Sample classification and pooling according to the RF and ACPA status (positive or negative); B) Immunodepletion of the pools; C) Mass spectrometry analysis by iTRAQ labelling (in duplicate).


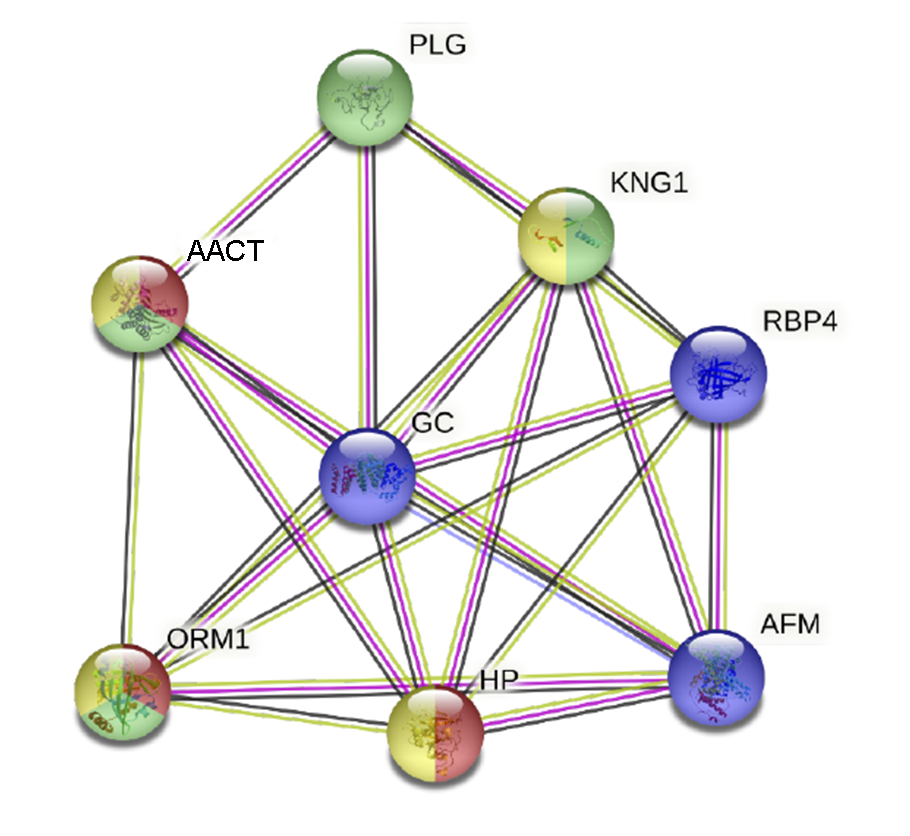


**Supplementary Figure S2.** Pathway analysis of the differential proteins verified by targeted MRM analysis.


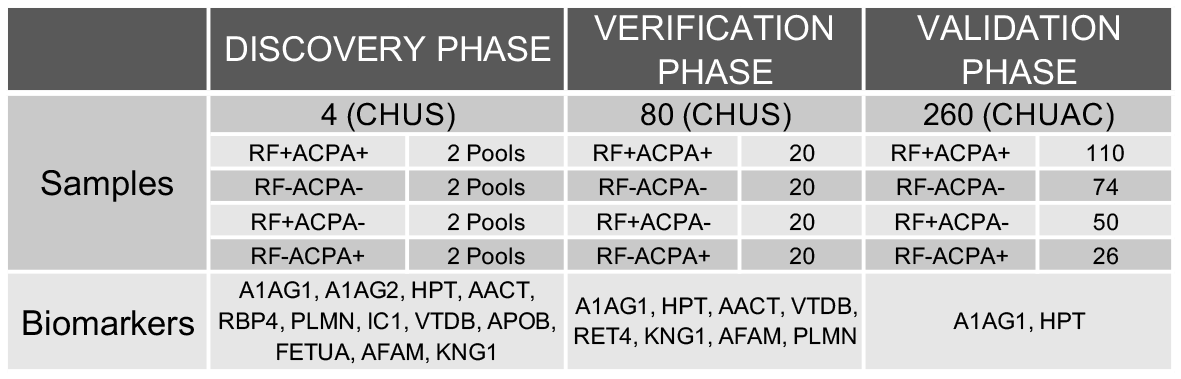


**Supplementary Figure S3.** Summary of the 3-step proteomic pipeline followed for the discovery and validation of RA markers.
